# Prediction and Production of Simple Mathematical Equations: Evidence from Visual World Eye-Tracking

**DOI:** 10.1371/journal.pone.0130766

**Published:** 2015-07-08

**Authors:** Florian Hintz, Antje S. Meyer

**Affiliations:** 1 Max Planck Institute for Psycholinguistics, Nijmegen, The Netherlands; 2 International Max Planck Research School for Language Sciences, Nijmegen, The Netherlands; 3 Donders Institute for Brain, Cognition, and Behavior, Radboud University, Nijmegen, The Netherlands; Leiden University, NETHERLANDS

## Abstract

The relationship between the production and the comprehension systems has recently become a topic of interest for many psycholinguists. It has been argued that these systems are tightly linked and in particular that listeners use the production system to predict upcoming content. In this study, we tested how similar production and prediction processes are in a novel version of the visual world paradigm. Dutch speaking participants (native speakers in Experiment 1; German-Dutch bilinguals in Experiment 2) listened to mathematical equations while looking at a clock face featuring the numbers 1 to 12. On alternating trials, they either heard a complete equation ("three plus eight is eleven") or they heard the first part ("three plus eight is") and had to produce the result ("eleven") themselves. Participants were encouraged to look at the relevant numbers throughout the trial. Their eye movements were recorded and analyzed. We found that the participants' eye movements in the two tasks were overall very similar. They fixated the first and second number of the equations shortly after they were mentioned, and fixated the result number well before they named it on production trials and well before the recorded speaker named it on comprehension trials. However, all fixation latencies were shorter on production than on comprehension trials. These findings suggest that the processes involved in planning to say a word and anticipating hearing a word are quite similar, but that people are more aroused or engaged when they intend to respond than when they merely listen to another person.

## Introduction

An important issue in psycholinguistics is the relationship between the language production system and the language comprehension system. Although language users draw upon both systems when communicating with one another, the two systems have mainly been studied independently of each other. Recently, however, a growing number of researchers have advocated the view that production and comprehension are tightly integrated [1–4]. The discussion of the production-comprehension interface has often focused on prediction. Research on language comprehension has established that readers and listeners often anticipate upcoming information [5–8] and that anticipation contributes to the speed with which they comprehend language. It is often assumed that predicting a word during comprehension is basically the same process as planning to say a word aloud. Scientists who argue for an integration of the comprehension and the production systems have also suggested that speakers, predict their own utterances and compare these predictions to the actual outcomes [9].

In the current study, we explored the similarity of word prediction during comprehension to word production using a novel version of the visual world paradigm [10]. Although there is correlational evidence for the involvement of production-based mechanisms in language comprehension, to our knowledge no study has directly compared the behavioral consequences of word prediction and word planning. We first summarize the key characteristics of word production and prediction processes. We then turn to recent proposals that suggest an integration of production and comprehension (i.e., prediction-by-production accounts) and discuss relevant empirical findings. Finally, we report two eye-tracking experiments in which we compared participants' eye movements reflecting their preparation to speak to eye movements reflecting their prediction of an upcoming word.

### Word Production

Various models of word production have been proposed [11–14]. Although they differ in their assumptions about the representations accessed when words are produced and about the processes involved, they agree that word production involves three main steps: speakers decide which concepts to refer to, select suitable words from the mental lexicon, and build up the corresponding word forms. For instance, the model proposed by Levelt, Roelofs, and Meyer [14] assumes three stages: conceptual preparation, lemma selection, and word form encoding (see also [15, 16]). The last-mentioned stage includes morphological, phonological, and phonetic encoding. During conceptual preparation, the speaker decides which concepts to encode. From the conceptual level, activation spreads to grammatical word units (lemmas), which are selected and ordered during lemma selection. Lemma selection is followed by the retrieval of the corresponding morphemes and phonological segments. The retrieved segments are combined into syllables. Based on the syllabified phonological representation a phonetic representation is created and, finally, articulatory commands are generated and executed. Speakers monitor their speech planning at the conceptual and phonological level and their overt speech for accuracy and appropriateness.

### Word Prediction

The brain has sometimes been said to be essentially a "prediction machine" [17–20] and many authors have proposed that prediction plays an important in language comprehension [21–23]. This view is well supported by experimental evidence. For instance, using recordings of event-related brain potentials, it was shown that semantic/conceptual information about upcoming language can be predicted [7, 24, 25] as well as the grammatical gender of words [8, 26]. To give a final example, there is experimental evidence indicating that listeners can predict the phonological forms of upcoming words [6, 27]. Altmann and Mirković [28] suggest that the comprehension system is "maximally incremental" in the sense that "it develops the fullest interpretation of a sentence fragment at each moment of the fragment’s unfolding" and at all possible levels (p. 604).

### Prediction-by-Production

Many authors have considered the possibility that predictions during comprehension are generated by mechanisms drawing upon knowledge also involved in speech production [8, 29–31]. This view has been explicitly implemented in two recent integrative frameworks. Dell and Chang [1] developed a model of sentence production, comprehension, and language acquisition where predicting the next word of an utterance is akin to planning to produce that word (see also [32]). Similarly, Pickering and Garrod [3] proposed that predictions during comprehension can be driven by an associative route, which is grounded in the comprehension system and by a simulation route, which engages the production system. With regard to the latter route, the authors suggest that language users construct forward models during production and comprehension, to predict their own utterances and to predict upcoming utterances by other speakers. In both cases the "predictions are not the same as implemented production representations but easier-to-compute 'impoverished' representations" (p. 339). Pickering and Garrod propose that both routes (e.g., prediction-by-association and prediction-by-production) can be used flexibly to predict information during comprehension.

Dell and Chang's and Pickering and Garrod's frameworks have in common that they equate the activation of word knowledge during prediction for comprehension with the activation of word knowledge during word planning. That is, the anticipation of the meaning, grammatical characteristics, or phonological form of upcoming words is equated with the activation of this information for speaking.

### Empirical Evidence for Production-based Mechanisms in Prediction

Several studies have reported correlational evidence for the involvement of production-based mechanisms in prediction and hence for a link between the comprehension and the production systems. Federmeier and colleagues [24, 33] found a significant correlation between participants’ prediction-related ERP components and their production fluency, as measured in a verbal fluency task. Furthermore, Mani and Huettig [34] observed that the production vocabulary size of two-year old toddlers predicted the degree to which the toddlers anticipated upcoming target words. These studies provide indirect evidence supporting prediction-by-production. However, an important further step towards understanding the involvement of production-based mechanisms in prediction is to compare word prediction directly to word planning processes carried out under identical circumstances.

To that end, we used a novel version of the visual world paradigm, which has previously been used to study prediction during comprehension [5, 35, 36] and language production [37–39]. In this paradigm, participants' eye movements are recorded while they view displays (e.g., showing a boy, a cake, and other objects) and hear sentences ("the boy will eat the cake", [5]) or produce utterances referring to the display. The eye movements indicate when the participants direct their attention to different parts of the displays and can, for instance, reveal whether or not listeners anticipate specific words (e.g., look at the cake in the above example before it is actually mentioned). In our experiments, Dutch speaking participants looked at the picture of an analogue clock face featuring the numbers 1 to 12. On half the trials, they listened to recordings of a person solving simple mathematical equations including the numbers 1 to 12, saying for instance "drie plus vijf is acht" (three plus five is eight). On the remaining trials, the recording stopped after "is", and the participants had to supply the solution of the equation. Listening and speaking trials alternated. In both tasks participants were asked to fixate on the relevant numbers on the clock face. After having carried out the computation, participants could predict what the recorded speaker would say and they could initiate the word planning process for their own production of the result number. We were interested in the similarity of the eye movements related to these processes.

We used spoken mathematical equations as materials because they allow for tight experimental control of variables such as word frequency and semantic associations. Moreover, in these utterances the final word is entirely predictable from the preceding context, yet different from trial to trial. Evidently, mathematical equations are not produced very often in everyday life. However, they are grammatically well-formed utterances. Producing and comprehending equations undoubtedly relies on grammatical, lexical, and phonological processes that are also involved in processing other types of utterances, such as descriptions of events and scenes, and therefore can be used to investigate these processes. Indeed, this has been done in several earlier studies. For instance, Ferreira and Swets [40] used the production of equations to study the scope of advance planning in sentence production; Bock et al. [41], Korvorst et al. [42] and Kuchinsky et al. [43] used time telling to study the mapping of conceptual information onto linguistic structures; and Scheepers et al. [44, 45] used priming between complex sentences and mathematical equations to study the involvement of shared processes and representations in arithmetic and sentence processing. Here, we exploited the simplicity of the lexical content of equations and the predictability of the result numbers to assess the involvement of prediction in speech planning and comprehension.

We recorded the participants’ eye movements throughout the experiment. We expected that in both tasks participants would follow the instructions and fixate upon each of the numbers they heard soon after word onset. In the production task, they should compute the result as soon as they had heard the second number, direct their gaze to the corresponding number on the clock face, and produce the response. We expected that the participants would initiate the shift of gaze to the appropriate location as soon as they had derived the number concept (rather than after having completely planned the utterance) and that they would therefore begin to fixate the number some time before the onset of their response. This coordination of eye gaze and speech planning would allow them to look at the response number while retrieving the corresponding verbal expression, which may facilitate these linguistic encoding processes [46]. In the listening condition, the participants might simply follow the listener, i.e., fixate upon each of the three numbers after it has been named. Alternatively, they could anticipate the result by computing it in the same way as on production trials. This would be consistent with the view that listeners engage their production system when they listen to another speaker and use it to predict what the speaker will say next [1, 3]. If the participants carry out the same computations and engage the speech production system in the same way on production and comprehension trials, their eye movements should not differ between the two conditions. A third possibility is that the prediction of the result number on comprehension trials is based not only on the engagement of production-based processes but is also supported by fast associative processes [47, 48]. In that case, one might expect faster eye movements to the result numbers on comprehension than on production trials.

To anticipate the main results of Experiment 1, we found that the participants' eye movements on production and comprehension trials were very similar, and that, specifically, they anticipated the result numbers on comprehension trials. Experiment 1 was carried out with native speakers of Dutch, who listened to and completed utterances in their native language. In Experiment 2, we asked German-Dutch bilinguals to carry out the same production and comprehension tasks as in Experiment 1 in Dutch, their second language. Previous research has shown that even in highly proficient late bilinguals, linguistic processing is slower, and presumably more effortful, than in native speakers [49–52] (see also [53, 54]). The goal of Experiment 2 was to determine whether the participants would still anticipate the result number or whether, given the higher linguistic processing load, they would simply follow the recorded speaker.

## Experiment 1

### Method

#### Participants

Twenty-five native Dutch participants (five male; mean age = 22 years, *SD* = 3 years), mostly students of Radboud University Nijmegen, participated in the experiment. All participants had normal or corrected-to-normal vision and hearing. None reported any signs or a history of developmental speech disorders. One participant had to be excluded from the sample because s/he mentioned during the debriefing that s/he had been diagnosed with dyscalculia.

#### Ethics Statement

All participants signed informed consent beforehand and were paid for their participation. Ethical approval of the study was provided by the ethics board of the Social Sciences Faculty of Radboud University.

#### Materials and Design

We constructed 60 stimulus sentences. The sentences were simple mathematical equations including the numbers one to twelve, using addition and subtraction (e.g., 1 + 5 = 6, spoken Dutch sentence: "Een plus vijf is zes"). Repetitions of numbers (as in 2 + 2 = 4 or 6 − 3 = 3) were avoided. The 60 sentences were spoken at a normal speech rate and with normal intonation contour by a native female speaker of Dutch. Recordings were taken in a sound-shielded booth sampling at 44 kHz (16 bit resolution). A second version of each equation was created by manually cutting off the result number at the offset of "is". The complete versions of the equations served as comprehension items. The incomplete versions of the equations served as production items. The mean length of the comprehension recordings was 4680 ms (*SD* = 234 ms); the mean length of the production counterparts was 3860 ms (*SD* = 180 ms). Onsets and offsets of all words in the spoken equations were marked using Praat [55]. We designed the picture of a round clock face featuring the numbers from one to twelve in their customary positions.

All participants were presented with all 120 items. The experiment was divided into four equal blocks, divided by short pauses. A pseudo-random order of the trials was generated. The constraints on the randomization were that production and comprehension trials alternated, the production and the comprehension versions of a given equation occurred in different blocks, and that successive items did not have the same result number.

### Procedure

The experiment was administered using an EyeLink 1000 system (SR Research) sampling at 1000 Hz. Participants placed their heads on a chinrest facing the computer screen 75 cm from the screen. Participants were instructed via the computer screen and invited to ask clarification questions. The eye-tracking system was calibrated and then the experiment began.

At the beginning of each trial, a black dot was presented on a white background in the middle of the screen for one second. Participants were asked to fixate the dot. This served as drift correction and ensured that participants always fixated the same position at the beginning of a trial. Subsequently, the clock face appeared in the center of the screen at a 600 x 600 pixel resolution, coinciding with the onset of the spoken sentence. The clock face remained in view during the entire trial. The trial duration for comprehension trials was 6000 ms (composed of the duration of the recording, on average 4680 ms, and individual timeouts, on average 1320 ms). The trial duration for production trials was 5500 ms (composed of the incomplete recordings, on average 3680 ms, individual timeouts of on average 1320 ms and 500 ms for participants' oral response). The participants were instructed to listen to the utterances and provide the result number on every second trial. They were also instructed to move their eyes to the numbers mentioned by the speaker as quickly as possible. This instruction was needed because a pilot study had shown that without such instruction participants would often fixate the center of the screen throughout the trial. Participants' responses to the production trials were recorded and coded during the experiment. The experiment lasted approximately 20 minutes.

### Data Coding and Dependent Variables

We excluded comprehension trials from the analysis on which participants uttered the result number by mistake (34 trials in total, <1% of all comprehension trials). Participants' speech onsets on production trials were hand-coded using Praat [55]. As the participants could begin to compute the result as soon as they had heard the second number of the recording, we defined the speech onset latency as the time period between the onset of that number and the onset of the participant's response. Production trials were excluded when the response was incorrect or the speech onset latency deviated by more than 2.5 standard deviations from the participant's mean onset latency (66 trials in total, <1% of all production trials).

For the eye movement analyses, the data from the participants' left or right eye (depending on the quality of the calibration) were analyzed and coded in terms of fixations, saccades, and blinks, using the algorithms provided in the EyeLink software. To determine how long each number was fixated for, regions of interests (90 x 90 pixels) were defined around each of the three numbers relevant for each equation. Two time windows were defined for the analyses: The time window for fixations to the first number started at the onset of the recording and lasted until the onset of the second number (on average 1686 ms, *SD* = 135 ms). The time window for the second number and result number started at the onset of the second number and ended at the offset of "is" (on average 1957 ms, *SD* = 144 ms). Note that this window only included fixations preceding the onset of the third number (i.e. predictive fixations in the comprehension condition, and fixations related to the preparation of the response in the production condition). For the statistical analyses we summed the fixation durations in the critical time windows and log-transformed the resulting total fixation durations.

We also calculated the fixation latencies for the first, second, and result numbers. As it takes about 200 ms to program and launch a saccadic eye movement [56], we consider the fixation latency to be the onset of the first fixation to a region of interest with a latency of 200 ms or more after the onset of the relevant time window (i.e., the onset of the utterance for fixations to the first number, and the spoken onset of the second number for fixations to the second and third number). Fixation latencies were log-transformed before analysis.

### Results

The analysis of the participants' speech onset latencies on production trials showed that they produced the result slightly later (by 85 ms) than the recorded speaker (means: 2128 ms, *SD* = 316 versus 2043 ms, *SD* = 200). This close match in the latencies is important because it facilitates the comparison of the participants’ eye movements in the production and comprehension conditions.

**Fig 1:**
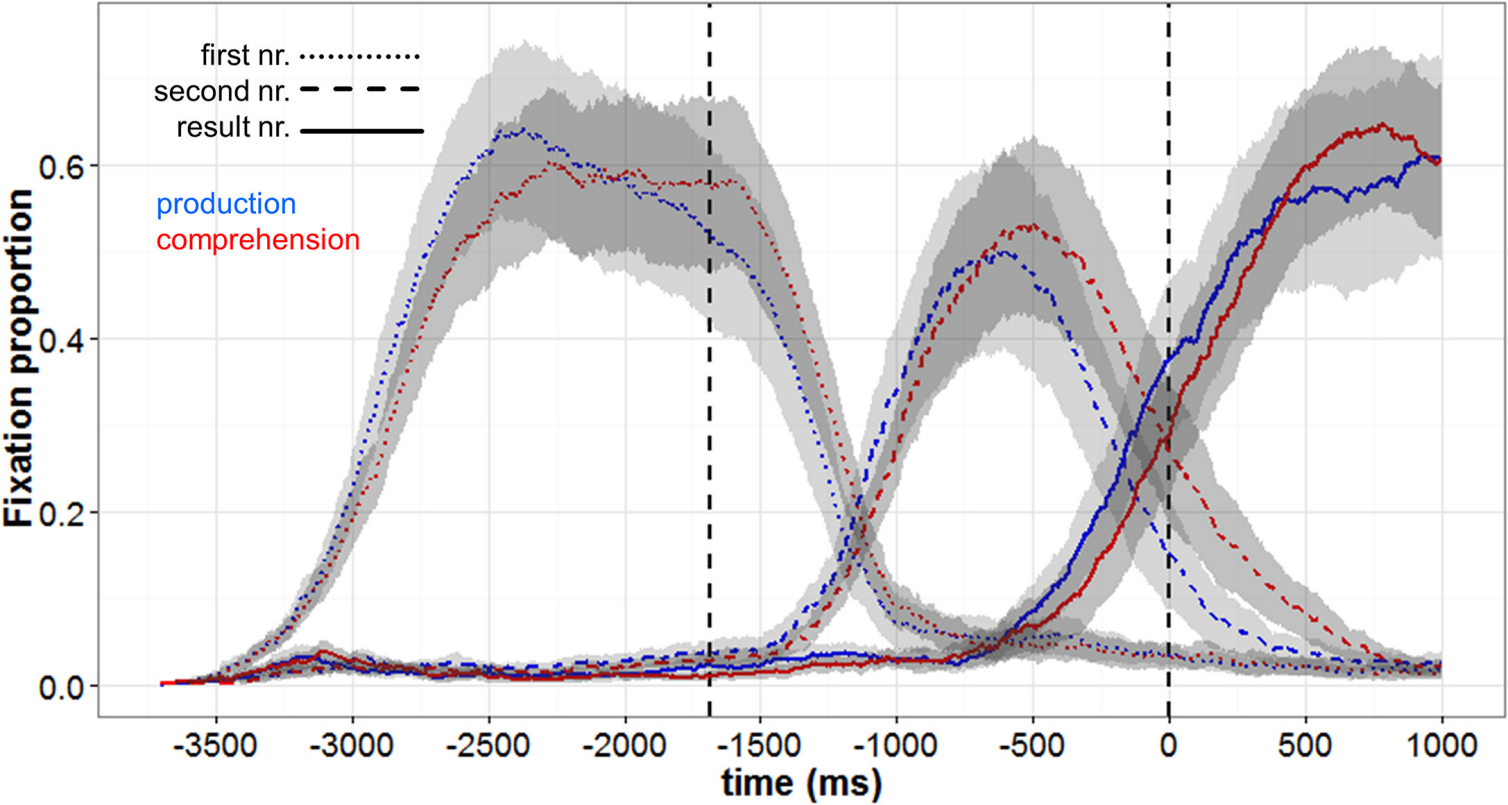
Fixation proportions in Experiment 1. The graph plots participants' average fixation proportions to first (dotted lines), second (dashed lines) and result number (solid lines) for production (blue) and comprehension (red) conditions. Fixations are plotted backwards from the offset of "is" in the recordings (time zero) to the onset of the first number. The first vertical dotted line represents the average onset of the second number. The areas shaded in gray represent the space in between the lower and upper bounds of the 95% by-participant confident intervals.

Fig 1 shows a time-course graph plotting the proportions of fixations to the first, second, and result number on production (blue) and comprehension (red) trials. Fixations are plotted backwards from the offset of "is" in the recording (time zero) to the onset of the first number. The vertical line, 1686 ms before time zero, indicates the average onset of the second number. Recall that each participant heard a given equation twice, once as a production version and once as a comprehension version. The fixation proportions indicate the proportion of trials (out of all relevant trials) on which participants fixated the first (dotted lines), second (dashed lines) and result number (solid lines), respectively, at that moment in time. We computed by-participant confidence intervals (95%) for each of the average fixation lines at every sampling step (1ms) to indicate the variation in participants’ fixation behavior. The area between the lower and upper bounds is shaded in light gray for production trials and in dark gray for comprehension trials. The graph shows that soon after utterance onset participants began to fixate the first number mentioned in the recording. Shortly after the onset of the second number, they stopped looking at the first number and started fixating the second number. Participants' likelihood of fixating the result number increased already before the offset of "is", that is, well before the result was named by the recorded speaker or before participants named it themselves. This behavior reflects that participants had calculated the solution of the equations. In the production condition participants began to look at the result number around one second after the onset of the second number, roughly 600 ms prior to the offset of "is". In the comprehension condition, fixations to the result number began about 100–200 ms later, but well before the onset of the spoken result number; on average, the participants' gaze landed on the result number 274 ms before its spoken onset.

Log-transformed total fixation durations for the time period between the onset of the second number and the offset of 'is' in the recording were analyzed using linear mixed-effects regression models in R ([57]; using the lme4 library, [58]). Mixed-effect models allow for simultaneous inclusion of participants and items as random factors [59]. The full model included the fixed effect of Condition (comprehension vs. production) and the maximal possible random effects structure [60], consisting of random intercepts and slopes for Condition by participant (N = 24) and item (N = 60). This model was compared to the same model without the fixed effect Condition using a likelihood test. Including Condition improved the model fit significantly, χ^2^ (2) = 9.091, *p* = .003. The full model revealed that, during the critical time window, participants looked more at the result numbers in the production as compared to the comprehension condition (production mean = 233 ms, *SD* = 253 vs. comprehension mean = 184 ms, *SD* = 240), *β* = -.638, *SE* = .196, *t* = -3.253 (*t* > |2| were considered significant; [59]). This result is complemented by the analysis of the fixation latencies, which showed that the participants' mean fixation latency for the result number was on average 143 ms shorter on production trials (1628 ms, *SD* = 598 ms) than on comprehension trials (1771 ms, *SD* = 673 ms). Log-transformed fixation latencies were submitted to a mixed effect model which was, apart from the dependent variable, identical to the model used for the fixation duration analysis. The model revealed the statistical robustness of the effect of Condition (*β* = -.07, *SE* = .024, *t* = -2.92; χ^2^ (2) = 7.762, *p* = .005).

Although the participants' eyes landed somewhat later on the result number on comprehension than on production trials, there is strong evidence that the participants anticipated the result numbers on comprehension trials instead of following the speaker. This can be appreciated by considering the fixation latencies measured from the onset of the spoken result number. Given that it takes at least 200 ms to recognize a spoken word [61] and a further 200 ms to initiate and launch a saccadic eye movement [56], saccades triggered by the spoken result number must have latencies of at least 400 ms. The observed average fixation latency was -284 ms. Thus, the participants began to look at the result number well before it was named by the recorded speaker. This was true for all speakers, with the slowest participant having a mean fixation latency of 150 ms.

In a supplementary analysis, we explored whether the participants' performance in the two tasks changed over the course of the experiment. To that end, we split the sequence of experimental trials into five blocks of 24 trials each and computed each participant's average fixation latency for the result numbers on comprehension and production trials. As one might expect, the participants' latencies were longer in the first block than in subsequent blocks. There was no consistent performance change across blocks 2 to 5. An analysis including block as an additional variable yielded no interaction with other variables.

We also compared fixation durations and fixation latencies between production and comprehension conditions for first and second number fixations (first number means: production = 892 ms, *SD* = 478 ms vs. comprehension = 833 ms, *SD* = 457; second number means: production = 501 ms, *SD* = 360 ms vs. comprehension = 547 ms, *SD* = 381). The analysis of fixation durations revealed no difference between the two conditions (first number: *β* = -.14, *SE* = .1, *t* = -1.353; χ^2^ (2) = 1.833, *p* = .176; second number: *β* = .224, *SE* = .186, *t* = 1.204; χ^2^ (2) = 1.434, *p* = .231). However, the analyses of the fixation latencies showed that the participants' gaze landed earlier on the first and on the second number on production trials (first number mean = 1831 ms, SD = 317 ms; second number mean = 792 ms, *SD* = 395 ms) compared to comprehension trials (first number mean = 1904 ms, *SD* = 347 ms; second number mean = 904 ms, *SD* = 493 ms; first number: *β* = -.037, *SE* = .013, *t* = -2.9; χ^2^ (2) = 8.004, *p* = .004; second number: *β* = -.118, *SE* = .022, *t* = -5.3; χ^2^ (2) = 19.689, *p* < .001). Thus, the participants were overall faster to react to the spoken input when they prepared for a response than when they merely listened to the recorded speaker.

### Discussion

In Experiment 1, we investigated how similar word prediction and word production processes are. We analyzed and compared eye movements reflecting participants' preparation to produce a word (the result of a simple mathematical equation) and eye movements reflecting their prediction of the same word being produced by a recorded speaker. In both conditions, we observed that the participants fixated upon the first two numbers of the equation in the order of mention, as they had been instructed to do, and then shifted their gaze to the result number. In both conditions, the shift of gaze to the result number occurred before the result number was spoken. Thus, in the production condition, the participants computed the result and then directed their gaze to the corresponding number and began to speak slightly afterwards. The average time eye-speech lag, i.e., the time between the onset of the fixation upon the result number and the onset of speech, was 490 ms (*SD* = 581). This substantial lag suggests that the participants initiated the saccade to the result number as soon as they had computed the result concept and carried out most of the linguistic planning of their utterance after the shift of gaze. We correlated the fixation latencies to the result numbers (measured from the spoken onset of the second number) with the eye-speech lags and found a moderate correlation *r* = .31 (*p* < .001 across 1240 production trials). Thus, speech latencies were faster on trials on which participants had looked at the target earlier. This correlation suggests that overlapping processes were engaged when participants planned the eye movement to the response number and when they subsequently planned the naming response.

Overall, the participants' eye movements in the two conditions were similar, suggesting that the cognitive processes occurring up to the overt articulation of the result number were similar as well. Although the similarity of the eye movements in the two conditions is striking, the analyses did reveal significant differences between the two conditions in the fixations to the three numbers. As shown in Table 1, the participants fixated the numbers earlier on production than on comprehension trials. This held not only for the result number, but also for the first two numbers, which were produced by the recorded speaker. As participants initiated the gaze to the result number earlier in the production condition than in the comprehension condition, the total duration of fixations to the result number was also longer. This was not the case for the first and second number, where fixations both began and ended earlier in the production than in the comprehension condition. Thus, it appears that participants were overall more engaged or aroused when they planned to speak than when they merely listened to the recorded speaker.

**Table 1:** Mean fixation durations and mean fixation latencies for production and comprehension conditions in Experiment 1 and 2.

	Experiment 1				Experiment 2			
	Fixation duration (ms)		Fixation latency (ms)		Fixation duration (ms)		Fixation latency (ms)	
	*speak*	*listen*	*speak*	*listen*	*speak*	*listen*	*speak*	*listen*
First number	892 (SD = 478)	833 (SD = 457)	1831 (SD = 317)	1904 (SD = 347)	863 (SD = 490)	836 (SD = 501)	1912 (SD = 371)	1959 (SD = 389)
Second number	541 (SD = 360)	548 (SD = 381)	792 (SD = 395)	904 (SD = 493)	454 (SD = 365)	494 (SD = 384)	970 (SD = 583)	1089 (SD = 679)
Result number	233 (SD = 253)	184 (SD = 241)	1628 (SD = 598)	1771 (SD = 673)	197 (SD = 237)	156 (SD = 216)	1699 (SD = 660)	1880 (SD = 687)

As the participants fixated the numbers earlier on production than on comprehension trials, one might expect that they would also produce the result number earlier than the recorded speaker. However, we observed the opposite, with participants taking slightly longer to produce the result than the recorded speaker. Recall, however, that the prerecorded speaker had been asked to read the equations at a moderate pace; hence the comparison of her speech onset latencies to those of the group of participants is not informative.

## Experiment 2

In Experiment 2, we used the same materials and design as in Experiment 1, but tested non-native speakers of Dutch. They were German students of the Radboud University with intermediate knowledge of Dutch. The goal of that experiment was to determine whether we could replicate the two main findings of Experiment 1, namely, first, that participants were faster to direct their eyes to the relevant stimuli on production than on comprehension trials, and, second, that they would predict the result numbers on comprehension trials. There is ample research demonstrating that lexical access is delayed in late bilingual individuals (as compared to native speakers), even at high levels of proficiency [49–52]. We expected that due to delayed lexical access the fixation latencies on the first two numbers would be longer than in Experiment 1. In addition, we reasoned that as the task would overall be somewhat more demanding for the non-native than for the native speakers, the non-native speakers might refrain from predicting the result numbers but simply follow the recorded speaker. This would result in later shifts of gaze to the result number on comprehension trials compared to production trials.

### Method

#### Participants

Twenty-four participants (6 male; mean age = 25 years, SD = 3 years) took part in Experiment 2. All were late German-Dutch bilinguals and students of the Radboud University. All had received class-room instruction in Dutch language and, at the time of participation, had been regularly speaking Dutch for at least six months (on average 43 months). They rated their proficiency as intermediate (3 on a five-point scale ranging 1 to 5). Note that numerals 1 to 12 are cognates in German and Dutch. Hence even for beginning speakers for Dutch, the task was not challenging. All participants had normal or corrected-to-normal vision and hearing. None reported any signs or a history of developmental speech disorders. All participants gave informed consent before the experiment and were paid for their participation.

#### Materials and Procedure

The instructions were translated into German. Apart from that, materials and procedure were identical to Experiment 1. Thus, the participants were asked to carry out the tasks in their second language.

### Results and Discussion

As in Experiment 1, we excluded production trials on which participants gave incorrect responses or their latency was more than 2.5 SD above their mean (57 trials; < 1%). We also excluded comprehension trials where participants uttered the result number by mistake (6 trials; < 1%). We calculated participants' naming latencies on production trials in the same way as in Experiment 1. The non-native speakers took on average 112 ms longer to start producing the result number than the recorded speaker. This is slightly longer (by 27 ms) than the time taken by the native speakers of Experiment 1.

**Fig 2:**
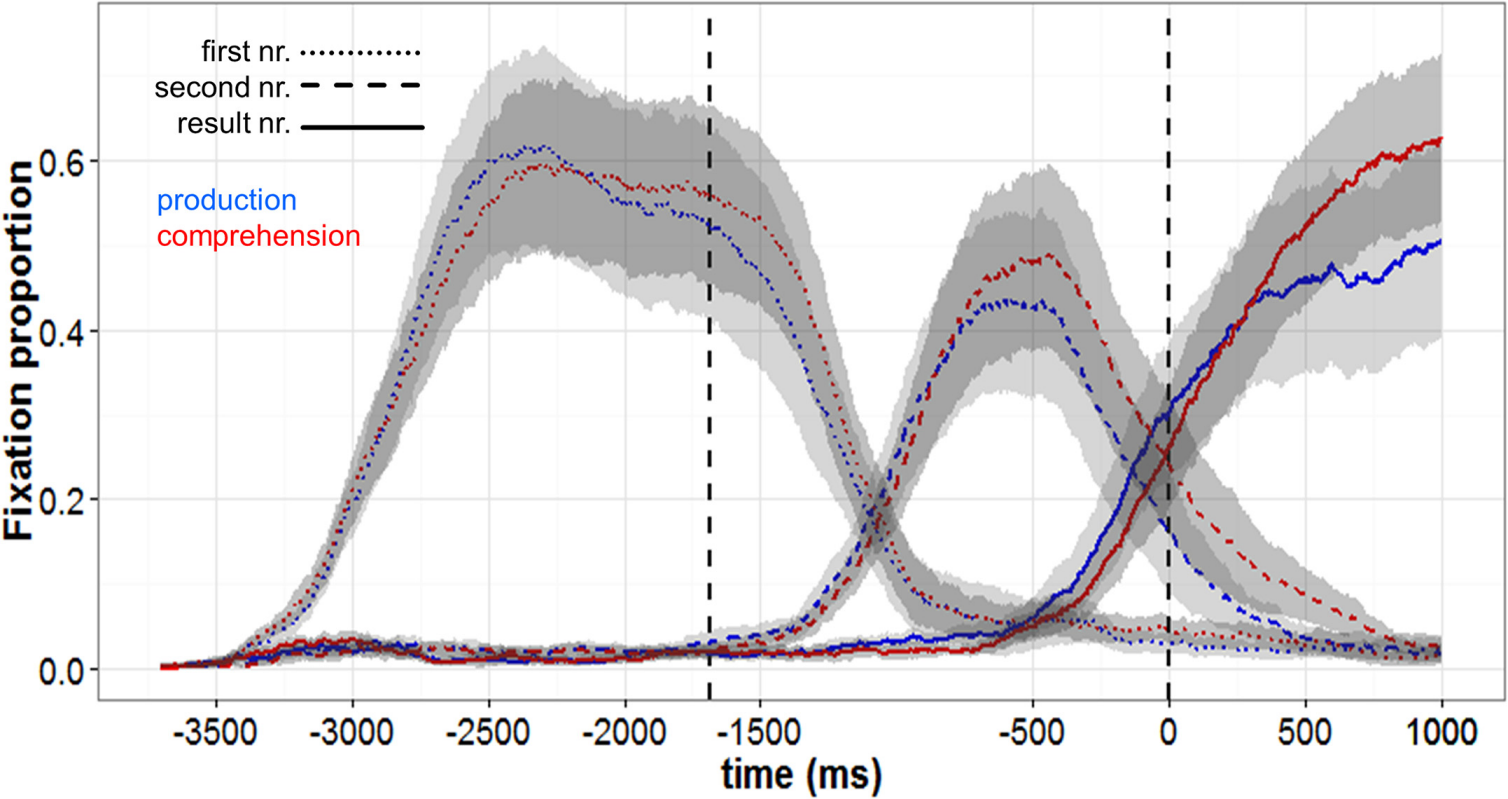
Fixation proportions of the German-Dutch bilinguals in Experiment 2. The data are plotted in the same way as for Experiment 1: Average fixation proportions to first (dotted lines), second (dashed lines) and result number (solid lines) for production (blue) and comprehension (red) conditions are shown. Fixations are plotted backwards from the offset of "is" in the recordings (time zero) to the onset of the first number. The first vertical dotted line represents the average onset of the second number. The areas shaded in gray represent the space in between the lower and upper bounds of the 95% by-participant confident intervals.

Fig 2 shows the average proportions of fixations to the first, second, and result number across the average trial plotted in the same way as for Experiment 1. As can be seen in Table 1, the non-native speakers were somewhat slower to fixate the three numbers than the native speakers, but apart from this expected difference, the results for the two groups of participants were very similar. In both the comprehension and the production condition, the participants first looked at the first and second number, in the order of mention, and then at the result number. As in Experiment 1, participants looked earlier at the relevant numbers on production than on comprehension trials. However, on comprehension trials they still looked at the result number before it was produced by the prerecorded speakers; the average lag was 197 ms. In other words, they anticipated the result numbers, as the native speakers of Experiment 1 had done.

The statistical analyses, carried out in the same way as for Experiment 1, confirmed that the results of Experiment 1 were replicated. As in Experiment 1, total fixation durations upon the first and second number did not differ between production and comprehension conditions (see Table 1, for means; first number: *β* = -.062, *SE* = .121, *t* = .711; χ^2^ (2) = .518, *p* = .472; second number: *β* = .212, *SE* = .23, *t* = .922; χ^2^ (2) = 1.669, *p* = .197), but there was a significant difference for the result number, with longer total fixation durations on production than on comprehension trials (*β* = -.575, *SE* = .139, *t* = -4.129). The comparison between the full model and a model that did not include the fixed factor Condition confirmed the better fit of the former (χ^2^ (2) = 13.35, *p* < .001). The fixation latencies to all three numbers were significantly shorter on production than on comprehension trials, with the difference being most pronounced for the result number (first number: *β* = -.026, *SE* = .011, *t* = -2.4; χ^2^ (2) = 5.377, *p* = .02; second number: *β* = -.091, *SE* = .023, *t* = -3.95; χ^2^ (2) = 12.914, *p* < .001; result number: *β* = -.0103, *SE* = .0389, *t* = 2.66; χ^2^ (2) = 6.917, *p* = .009). This pattern suggests that the participants were more alert on speaking than on listening trials.

We examined whether the participants' performance changed over the course of the experiment. The sequence of trials was split into five blocks of 24 trials each and computed each participant's average fixation latency for the result numbers on comprehension and production trials. The latencies were longer in the first block than in subsequent blocks, but there was no further consistent performance change across the following blocks. In all blocks, fixation latencies were shorter on production than on comprehension trials. An analysis including block as an additional variable yielded no main effect of this variable.

Finally, we examined whether all participants anticipated the result number on comprehension trials, i.e. had average latencies, measured from the onset of the result number, below 400 ms. This was the case for 22 of the 24 participants. The remaining participants had latencies of 351 and 442 ms, respectively, but were also much slower than average (by 1011 ms and 730 ms, respectively) to fixate the second number in the equation. Thus, these participants processed the equations very slowly. Whether they did not aim to anticipate the result numbers or simply did not complete the mental computation before the numeral was produced by the recorded speaker cannot be determined.

In sum, the results of Experiment 2 closely replicate those of Experiment 1. The non-native speakers of Dutch were only slightly slower than the native speakers to fixate the relevant numbers on the clock face and to produce the result number, and on both production and comprehension trials their eyes landed on the result number before the numeral was produced. Thus, on comprehension trials they predicted the last word of the utterance, as the native speakers of Experiment 1 had done. Individual analyses of the difference between their first fixation upon the target on comprehension trials and the onset of result number in the recording showed that there were two participants with long positive lags (351 ms, 442 ms).

Most likely, the results obtained for the two groups of speakers were very similar because the difficulty of the task did not differ much for them; in other words, contrary to our expectation, the non-native speakers found the tasks almost as easy as the native speakers did. Evidently, on the basis of these data no claims can be made about prediction in non-native language comprehension in general (but see [62, 63]). The present results do, however, show that the main results—prediction of upcoming result numbers and earlier fixations of the numbers on production than on comprehension trials—can readily be replicated.

## General Discussion

Recent theories of language comprehension propose that listeners employ production-based mechanisms to anticipate upcoming language [1–3]. In the current study, we tested this claim by comparing participants' planning of a word with their prediction of the same word being produced by a recorded speaker—both processes carried out under identical circumstances. In two experiments, participants listened to mathematical equations containing the numbers 1 to 12 while looking at an analogue clock face. We instructed participants to fixate the numbers on the clock face mentioned by the speaker as quickly as possible. On alternating trials, they listened to the entire equation or produced the result number themselves when the recording stopped after "is". In Experiment 1, the participants were native speakers of Dutch, and in Experiment 2 they were native speakers of German using Dutch as their second language. We tested a strong prediction of prediction-by-production accounts of processing: If word prediction is identical to internal word production, we should observe identical behavioral consequences caused by both processes. We measured participants' eye movements preceding their own speech onsets and preceding the onset of the spoken numerals in the recording. In both experiments, we observed shifts in eye gaze to the result numbers prior to the respective word onsets. In both experiments, however, we also observed statistically significant differences in the fixation latency and duration for the result numbers between the conditions: The participants began to fixate the result number earlier and consequently spent more time fixating it on production trials than on comprehension trials. Moreover, participants were also faster to locate the first and second numbers of the equations on production than on comprehension trials.

These results allow for three conclusions. First, in the comprehension condition, the participants predicted the last word of the spoken utterances. The task did not require them to do so; they could have waited until the recorded speaker named the result numbers and then direct their gaze towards them. However, instead of following the recorded speaker, the participants computed along with her, predicting what she would say. In evaluating this finding it is important to keep in mind that the participants knew that their eye movements were recorded and that they had been asked to fixate the relevant numbers. They were not asked to anticipate what the recorded speaker might say. Nevertheless, it is possible that participants felt that looking at each number as soon as possible and anticipating the results on comprehension trials would be desirable. It should also be kept in mind that in our experiments comprehension and production trials alternated. There may have been a transfer effect between trial types, and participants may have been more likely to engage in the mental computation of the results on comprehension trials than they would be if, for instance, production and comprehension trials appeared in different blocks [64]. Further work is needed to determine under which conditions listeners engage in which kinds of predictions [23]. Our study demonstrated that under the conditions we created, the participants anticipated the last word of the spoken utterances.

Second, judging from the participants’ eye movements, the cognitive processes occurring on production and comprehension trials, up to the time when the result numbers were produced or heard, were very similar. This can best be appreciated by comparing the fixation proportions to the three numbers of the equations shown in Figs 1 and 2. One may say that the similarity of the eye movements and the underlying cognitive processes is hardly surprising given the similarity of the production and comprehension tasks. However, as noted, the participants did not have to predict the result numbers and direct their eyes towards them in anticipation of the speaker. They elected to do so, and the cognitive processes involved in computing the result number are very likely to have been the same as those engaged in computing the result number for overt articulation. Thus, our results are in line with the view that the participants engaged largely the same processes on comprehension and on production trials. As noted in the Introduction, it has been proposed that predictions during comprehension might be based on fast associative processes, which are not engaged when speakers prepare utterances [47, 48]. If such associative processes played a major role in our task, one might have observed faster eye movements to the result numbers on comprehension than on production trials. However, as discussed further below, we found the opposite pattern. Thus, our results do support the view that prediction on comprehension trials was based on processes that were engaged on production trials.

Third, we found a consistent difference in the participants' fixation latencies to the three numbers. They looked earlier at the first and second number mentioned by the recorded speaker and they directed their gaze earlier to the result number when they had to produce it than when they merely listened to the recorded speaker. This unexpected result suggests that the participants were more engaged or aroused when an overt response was required than when they merely listened to the other person. The fixation latency difference between the production and comprehension conditions was more pronounced for the result number than for the first and second numbers, probably because the arithmetic operation required to compute the result benefitted more from a higher activation level than the processes involved in listening to the utterances and locating the numbers on the clock face. In short, the participants carried out very similar cognitive processes on production and comprehension trials, but did so with a higher degree of engagement or arousal when an overt response was required, and perhaps *because* an overt response was required. Perhaps a different pattern of results is seen when participants have to provide an overt response on comprehension trials as well (e.g., indicating whether or not the response given by the recorded speaker is correct). In that case, eye movements to the result number might be equally fast on production and comprehension trials; or they might even be faster on comprehension trials if associative processes are engaged during comprehension. The effects of various comprehensions task can be addressed in future research.

The current project involved mathematical equations, and one may ask whether the results are indicative of the relationship between production and comprehension processes occurring when people comprehend and produce everyday utterances. This is an empirical issue, which could be investigated by comparing the eye movements of speakers describing scenes and events (saying, for instance, "the boy will eat the cake") to the eye movements of listeners hearing descriptions of the same scenes. As discussed earlier, there is a large body of evidence demonstrating that listeners predict upcoming parts of utterances, and it is highly likely that these predictions are based on conceptual and linguistic knowledge that is also assessed when people produce utterances. Yet, whether this knowledge is used in the same way and equally efficiently in speaking and listening is not known. To assess this issue it is necessary to compare the time course of conceptual and linguistic processes for production and comprehension in tasks that are as similar as possible. A methodological contribution of the present study is to demonstrate how this could be done.

It has often been proposed that speaking is more effortful than comprehending language. A number of reasons for this difference have been proposed, for instance that speakers must develop more complete representations than listeners and that the speakers must not only develop, but also monitor their speech plans for correctness and appropriateness for the communicative situation [65]. There is some evidence for the claim that speaking is indeed more demanding than listening, for instance from dual-task studies [66, 67], though the evidence is by no means unambiguous [68]. In any event, it is often assumed that the processes involved in speaking are inherently more complex than those involved in listening, which leads to a higher degree of felt cognitive effort. Our results do not allow us to decide whether or not this view is correct. They do suggest, however, that in addition to any inherent differences in the complexity of production and comprehension processes, motivational differences may also contribute to differences in experienced effort: People may be more aroused or attentive when they speak than when they listen, possibly for the simple reason that the results of their efforts are witnessed by others when they speak but not when they listen to language.

In sum, the present study illustrates how eye-tracking can be used to track the time course of some of the processes occurring when people listen to spoken sentences and anticipate upcoming words and when they prepare to say these words themselves. Our evidence suggests, first, that prediction during sentence comprehension and speech planning may indeed be closely related processes, and, second, that people are more active or aroused when they intend to complete another person's utterance than when they merely listen to the interlocutor.
